# Usefulness of lumbar puncture educational videos for older people with HIV

**DOI:** 10.3389/fdgth.2025.1508163

**Published:** 2025-02-13

**Authors:** Chhitij Tiwari, Keely Copperthite, Tia Morgan, Jonathan Oakes, Luigi Troiani, Chris Evans, Sonia Napravnik, Claire E. Farel, Monica M. Diaz

**Affiliations:** ^1^ Department of Neurology, University of North Carolina at Chapel Hill School of Medicine, Chapel Hill, NC, United States; ^2^ Institute for Global Health and Infectious Diseases, University of North Carolina at Chapel Hill School of Medicine, Chapel Hill, NC, United States; ^3^ Center for AIDS Research, University of North Carolina at Chapel Hill School of Medicine, Chapel Hill, NC, United States

**Keywords:** people with HIV, aging, neurological, lumbar puncture, video-based education

## Abstract

**Background:**

Video-based education offers opportunities to enhance patients' medical literacy and to reduce anxiety and hesitation for patients undergoing diagnostic procedures such as lumbar puncture (LP). Multiple studies centered on LP education have demonstrated that video-based education can reduce anxiety regarding possible adverse events, while increasing literacy regarding the procedure itself for clinical purposes. Our study sought to assess the impact of video-based education on knowledge of and willingness to undergo an LP among older people with HIV (PWH).

**Methods:**

We enrolled PWH age ≥ 50 years who regularly attend our Infectious Diseases clinic between March 3 and November 16, 2023. Participants watched a patient-centered educational video explaining the LP procedure and completed a questionnaire both pre- and post-video assessing demographics, general awareness and prior experience with an LP, specific knowledge, attitudes and perceptions toward an LP and willingness to undergo an LP.

**Results:**

Our study included 99 PWH with mean (standard deviation, SD) age of 58.8 (5.7) years, one-third females and 60% African American/Black race. After watching the video, participants were significantly more likely to correctly identify technical details of the procedure (excluding those who had previously had an LP, 83.7% pre-video vs. 95.9% post-video) and common complications of an LP; agree that LPs can result in back pain (*p* < 0.001) and headaches (*p* < 0.001). There was no significant difference in participants' willingness to undergo an LP for diagnostic or research purposes. Only 5% said that they would never have an LP under any circumstance after watching the video.

**Conclusions:**

Other educational interventions, such as in-person demonstrations or models, may help mitigate fears of LP. Our study provides important insight into the knowledge and perceptions of PWH when asked to undergo an LP and demonstrates that video-based education may not be sufficient to mitigate fears surrounding LP procedures, or a lack of interest or time for participating in an LP.

## Introduction

With recent advances in technology, the use of video-based educational interventions offers significant potential for improving patient care on a variety of outcome measures. Using portable handheld devices, such as cell phones or tablets, brief informative videos can be distributed to patients in a variety of healthcare settings ranging from inpatient wards to clinic waiting rooms (1–5). They can also be utilized to improve a variety of healthcare outcomes, including vaccination rates and patients' medical literacy (1, 2, 4).

Video-based education has also been explored in clinical contexts where procedures may be necessary for patient care. These patient-centered educational resources have been shown to decrease patient anxiety surrounding potential risks or discomfort related to various procedures, including lumbar punctures (LPs) and coronary angiography (6–8). This decrease in anxiety has been shown to be particularly beneficial in the context of obtaining consent for clinical procedures (8). Video-based education has also improved outcomes directly related to the procedures themselves. One study, for example, demonstrated increased adherence with bowel preparation for colonoscopies and higher rates of adenoma resection in patients who received virtual reality video education (i.e., a simulation of being physically present in the procedure room) compared with those who only received written and oral instructions (3).

Video-based education offers significant opportunities for improving patient care when an LP may be necessary for diagnosing neurological conditions in people with HIV (PWH), such as for HIV-related central nervous system (CNS) co-infections or opportunistic infections. A few select studies have been conducted that reflect the potential value of these videos among people without HIV. Multiple studies centered on LP education have demonstrated that video-based education can reduce anxiety regarding possible adverse events, while increasing literacy regarding the procedure itself for clinical purposes (7, 8). To date, however, no study has evaluated the potential impact of video-based education on patient willingness to undergo LPs for research purposes among people without HIV, nor among PWH in clinical or research settings.

To understand the impediments of implementing LPs for advancement of research on PWH, we proposed to determine the knowledge and willingness of middle-aged and older PWH to undergo an LP for research purposes. Our study aimed to assess the impact of video education on the knowledge and perceptions of older PWH on LPs by assessing their knowledge and anxiety regarding the procedure. We also sought to determine whether a patient-centered educational video on LPs would improve willingness to receive an LP for research purposes. We hypothesized that video education would increase knowledge, reduce anxiety, and increase willingness to undergo the procedure for research purposes.

## Methods

### Study participants and selection

The goal of this study was to assess the effect of video education on participants' willingness to undergo an LP procedure, either for diagnostic or research purposes. This was a sub-study from a cross-sectional parent study with the objective of determining if neuroinflammation may play a role in neurodegeneration. We completed a sub-study within the parent study that assessed knowledge of and willingness to undergo an LP before and after watching a brief instructional patient-centered video on an LP procedure. Participants were selected through convenience sampling. As part of the parent study, we enrolled male and female PWH who were 50 years of age or older and had attended one or more clinical visits at our clinic in the 12 months prior to recruitment. We excluded any patient (or their legal healthcare proxy if available) who was unable to provide verbal informed consent and those in the clinic who did not have HIV. HIV status was based on review of the clinical record that was reviewed prior to recruitment into the study. Participants were invited to participate by a research assistant if they met inclusion criteria before or after their scheduled clinic visit with their HIV provider. Data was collected from March 3, 2023, to November 16, 2023.

### Study procedures

Participants then completed the verbal informed consent process and completed a brief questionnaire administered by the research assistant assessing knowledge of LP procedures and willingness to undergo an LP for research purposes. As part of the consent process, participants were informed that they would not receive any direct benefits from the study. Participants then watched a brief 5-minute patient-tailored educational video on an electronic tablet explaining the LP procedure from *Your Practice Online*, an online library of patient education videos, that highlights indications for the procedure, the procedure process, risks and complications of an LP, among others (9). This video utilizes a combination of infographics, as well as 3D animations, to demonstrate the procedure and discuss the indications for an LP (9). The same brief questionnaire was then re-administered to the participant after watching the video. This video was selected based on ease of understanding from a patient perspective and brevity. After watching the video, the participant was also informed prior to the post-LP questionnaire that the LP may also take place in the upright seated position in addition to the lateral decubitus position that was shown in the video.

The questionnaire was adapted from the *Alzheimer's Disease Center Pre-LP Survey* that was administered to all enrolled participants (10). The questionnaire consisted of three parts: (1) demographics (age range, sex, self-identified race and ethnicity, educational level); (2) general awareness and prior experience with an LP; (3) specific knowledge and attitudes toward LP related to diseases, including what types of conditions an LP can help diagnose; perceptions of an LP; respondent's willingness to consider an LP for different purposes.

### Statistical analyses

We performed descriptive statistics of all data, including summary statistics for continuous variables and frequencies and percentages for all categorical variables. For demographic statistics, Fisher's exact test was used to determine differences in subgroups for participants who had undergone an LP previously vs. participants who had not. To contrast participant responses to the questionnaire pre- and post-video, we used McNemar's test for questions with two options (Yes and No), and Bhapkar's test for questions with four options (Yes, No, Don't Know, and Prefer Not to Answer). McNemar's test was used to contrast participant preferences (Yes and No) for undergoing an LP pre- and post-video. All statistical analyses were performed in RStudio (11).

## Results

Our study population included 99 PWH who completed the study from March 1, 2023, to November 16, 2023. The demographic characteristics of the participants, as well as their prior knowledge of and previous experiences with LPs, are displayed in Table 1. The mean age (standard deviation, SD) of the study participants was 58.8 years (5.69) with one-third females and 60% identifying as African American or Black race. Nearly half of participants had an LP in the past. Approximately 72% of participants had a history of frequent back pain (defined as once per week or more often), and approximately 20% of participants had frequent headaches, similarly defined. Further analysis of participant characteristics is provided in Table 2, with stratification based on prior history of LP. African American participants were less likely to have undergone a prior LP, while Caucasian participants were more likely to have undergone a prior LP; all other demographic characteristics were nonsignificant between subgroups.

**Table 1:** Characteristics of study participants (*N* = 99).

Characteristic	Overall [*n* (%)]
Age	Range 50–72
<55	30 (30.0%)
55–64	54 (55.0%)
65–74	15 (15.0%)
Sex	
Female	33 (33.3%)
Male	66 (66.7%)
Race	
African American	59 (60.0%)
American Indian/Native Alaskan	1 (1.0%)
Caucasian	36 (36.0%)
Other, please specify	3 (3.0%)
Ethnicity	
Hispanic/Latino	6 (6.1%)
Not Hispanic/Latino	90 (90.9%)
Unknown	3 (3.0%)
Highest Education Level	
Some high school, but no diploma	14 (14.0%)
High school diploma/GED	23 (23.0%)
Some college, but no degree	24 (24.0%)
Associate Degree	13 (13.0%)
Bachelor's Degree	16 (16.0%)
Master's Degree	8 (8.1%)
Professional degree (JD, MD, DO, PhD)	1 (1.0%)
Current Employment Status	
Disabled	31 (31.0%)
Full-time	31 (31.0%)
Part-time	10 (10.0%)
Prefer not to answer	2 (2.0%)
Retired	12 (12.0%)
Unemployed	13 (13.0%)
Previous LP	
Yes	47 (47.0%)
No	49 (49.0%)
Don't know	3 (3.0%)

**Table 2:** Participant characteristics, stratified by history of lumbar puncture.

Characteristic	Don't know (*N* = 3)^a^	No (*N* = 49)^a^	Yes (*N* = 47)^a^	*p*-value^b^
Age				0.5
< 55	0 (0%)	16 (33%)	14 (30%)	
55–64	2 (67%)	24 (49%)	28 (60%)	
65–74	1 (33%)	9 (18%)	5 (11%)	
Sex				>0.9
Female	1 (33%)	17 (35%)	15 (32%)	
Male	2 (67%)	32 (65%)	32 (68%)	
Race				0.013
African American	2 (67%)	35 (71%)	22 (47%)	
American Indian/Native Alaskan	0 (0%)	0 (0%)	1 (2.1%)	
Caucasian	0 (0%)	13 (27%)	23 (49%)	
Other	1 (33%)	1 (2.0%)	1 (2.1%)	
Ethnicity				0.7
Hispanic/Latino	0 (0%)	4 (8.3%)	2 (4.3%)	
Not Hispanic/Latino	3 (100%)	43 (90%)	44 (96%)	
Prefer not to answer	0 (0%)	1 (2.1%)	0 (0%)	
Unknown	0	1	1	
Highest educational level				0.3
Associates Degree	0 (0%)	3 (6.1%)	10 (21%)	
Bachelor's Degree	0 (0%)	7 (14%)	9 (19%)	
High school diploma/GED	0 (0%)	13 (27%)	10 (21%)	
Master's Degree	0 (0%)	5 (10%)	3 (6.4%)	
Professional degree (JD, MD, DO, PhD)	0 (0%)	1 (2.0%)	0 (0%)	
Some college, but no degree	1 (33%)	14 (29%)	9 (19%)	
Some high school, but no diploma	2 (67%)	6 (12%)	6 (13%)	
Current employment status				0.14
Disabled	2 (67%)	15 (31%)	14 (30%)	
Full-time	0 (0%)	16 (33%)	15 (32%)	
Part-time	0 (0%)	8 (16%)	2 (4.3%)	
Prefer not to answer	1 (33%)	0 (0%)	1 (2.1%)	
Retired	0 (0%)	5 (10%)	7 (15%)	
Unemployed	0 (0%)	5 (10%)	8 (17%)	
Place of birth				0.6
Inside the U.S.	3 (100%)	48 (98%)	45 (96%)	
Outside the U.S.	0 (0%)	1 (2.0%)	2 (4.3%)	

^a^ *n* (%).

^b^ Fisher's exact test.

### Descriptive analysis

The results of the analyses comparing pre-video and post-video responses are provided in Table 3. Excluding participants who had previously received an LP, 83.7% of participants were able to correctly identify a visual depiction of an LP being conducted pre-video; post-video, 95.9% were able to successfully identify the correct image. This improvement was not statistically significant. When asked to identify the correct region of the spinal cord for insertion of the lumbar needle, participants were significantly more likely to identify the correct region after watching the video; this analysis was also conducted after excluding participants who had previously had an LP (44.9% pre-video, 77.6% post-video, *p* < 0.01). All participants were significantly more likely to correctly ascertain what an LP generally entailed (*p* < 0.001) after watching the video. Participants were significantly more likely to correctly ascertain that LP could be used to diagnose meningitis (*p* < 0.001) and multiple sclerosis (*p* < 0.01). There was no significant difference in their choices, pre- and post-video, regarding whether an LP can be used to diagnose depression, stroke, skin diseases, or Parkinson's disease.

**Table 3:** Knowledge of LP pre- and post-LP educational video among all participants (*N* = 99).

Question		Yes (%)	No (%)	Don't know (%)	Prefer no answer (%)	*p*-value
LP is inserting a needle at the lower back to withdraw spinal fluid.	Pre	85	4	8	**2**	**<0.001**
	Post	98	1	0	0	
LP requires anesthesia (medications to make you sleepy).	Pre	20	51	25	**2**	**<0.001**
	Post	35	60	3	0	
LP can cause back pain.	Pre	49	15	32	**1**	**<0.001**
	Post	81	12	6	0	
After LP, a patient may have difficulty urinating or holding their urine.	Pre	8	34	55	**2**	**<0.001**
	Post	6	66	27	0	
LP causes severe complications, like being paralyzed or in a wheelchair.	Pre	30	27	39	**2**	**<0.001**
	Post	19	56	24	1	
LP (spinal tap) is a low-risk procedure and relatively safe.	Pre	61	15	21	**2**	**<0.001**
	Post	86	9	3	0	
The most common complication for LP (spinal tap) is a headache.	Pre	37	14	47	**1**	**<0.001**
	Post	79	10	10	0	
Meningitis can be diagnosed via LP.	Pre	65	34	N/A	**N/A**	**<0.001**
	Post	86	13	N/A	N/A	
Multiple sclerosis can be diagnosed via LP.	Pre	56	43	N/A	**N/A**	**<0.01**
	Post	75	24	N/A	N/A	
Depression can be diagnosed via LP.	Pre	3	96	N/A	N/A	0.21
	Post	8	91	N/A	N/A	
Stroke can be diagnosed via LP.	Pre	11	88	N/A	N/A	0.53
	Post	15	84	N/A	N/A	
Skin diseases can be diagnosed via LP.	Pre	7	92	N/A	N/A	0.33
	Post	12	87	N/A	N/A	
Parkinson's disease can be diagnosed via LP.	Pre	33	66	N/A	N/A	0.24
	Post	42	57	N/A	N/A	

Bold values are statistically significant, *p* < 0.05.

### Evolution of knowledge of LP procedures

After watching the video, participants were significantly more likely to agree that LPs can result in back pain (*p* < 0.001) and headaches (*p* < 0.001). Subjects were significantly less likely to select “Don't know” regarding whether LPs require general anesthesia (25.5% pre-video, 3.1% post-video, *p* < 0.001) and whether LPs can result in severe complications such as being wheelchair-bound (39.8% pre-video, 24.5% post-video, *p* < 0.001) or experiencing urinary incontinence (56.1% pre-video, 27.6% post-video, *p* < 0.001). Subjects were significantly more likely to agree after watching the video that LPs are low-risk and relatively safe (*p* < 0.001). After excluding subjects who preferred not to answer, there was no significant difference in how frightened subjects were, on a scale from 1 to 7, of receiving an LP.

Tables 4, 5 provide additional information regarding participants' knowledge of LP, with stratification based upon prior LP (Table 4) and no prior LP (Table 5). Participants who had a previous LP significantly improved in their knowledge of the procedure itself, including correctly identifying frequent side effects such as headache while avoiding identifying rare side effects. There was no significant change in their perceptions, pre- and post-video, regarding whether LP was a safe procedure (pre-video 72% of participants, post-video 83%; *p* = 0.23). These participants either correctly identified diagnostic purposes of an LP both before and after the video, or significantly improved in their awareness of its diagnostic uses.

**Table 4:** Responses of participants to questions of knowledge with previous LP experience (*N* = 47).

Question		Yes (%)	No (%)	Don't know (%)	Prefer no answer (%)	*p*-value
LP is inserting a needle at the lower back to withdraw spinal fluid.	Pre	91	2	4	2	**<0.001**
	Post	100	0	0	0	
LP requires anesthesia (medications to make you sleepy).	Pre	10	75	13	2	**<0.01**
	Post	21	74	4	0	
LP can cause back pain.	Pre	53	21	21	4	**<0.001**
	Post	83	9	6	0	
After LP, a patient may have difficulty urinating or holding their urine.	Pre	6	45	45	4	**<0.05**
	Post	4	64	32	0	
LP causes severe complications, like being paralyzed or in a wheelchair.	Pre	33	33	30	4	**<0.001**
	Post	23	51	26	0	
LP (spinal tap) is a low-risk procedure and relatively safe.	Pre	72	21	4	2	**0.23**
	Post	83	13	2	2	
The most common complication for LP (spinal tap) is a headache.	Pre	53	13	32	2	**<0.001**
	Post	89	4	6	0	
Meningitis can be diagnosed via LP.	Pre	72	28	N/A	**N/A**	**<0.01**
	Post	92	17	N/A	N/A	
Multiple sclerosis can be diagnosed via LP.	Pre	45	55	N/A	**N/A**	**<0.01**
	Post	72	28	N/A	N/A	
Depression can be diagnosed via LP.	Pre	0	100	N/A	N/A	>0.05
	Post	0	100	N/A	N/A	
Stroke can be diagnosed via LP.	Pre	9	91	N/A	N/A	>0.05
	Post	11	89	N/A	N/A	
Skin diseases can be diagnosed via LP.	Pre	9	91	N/A	N/A	>0.05
	Post	11	89	N/A	N/A	
Parkinson's disease can be diagnosed via LP.	Pre	26	74	N/A	N/A	**<0.05**
	Post	45	55	N/A	N/A	

Bold values are statistically significant, *p* < 0.05.

**Table 5:** Responses of participants to questions of knowledge without previous LP experience (*N* = 47).

Question		Yes (%)	No (%)	Don't know (%)	Prefer no answer (%)	*p*-value
LP is inserting a needle at the lower back to withdraw spinal fluid.	Pre	80	6	12	2	**<0.001**
	Post	98	2	0	0	
LP requires anesthesia (medications to make you sleepy).	Pre	28	31	39	2	**<0.001**
	Post	49	47	4	0	
LP can cause back pain.	Pre	47	10	43	0	**<0.001**
	Post	80	14	6	0	
After LP, a patient may have difficulty urinating or holding their urine.	Pre	8	24	67	0	**<0.001**
	Post	4	73	22	0	
LP causes severe complications, like being paralyzed or in a wheelchair.	Pre	27	22	49	2	**<0.001**
	Post	25	63	22	0	
LP (spinal tap) is a low-risk procedure and relatively safe.	Pre	49	10	39	2	**<0.001**
	Post	90	6	4	0	
The most common complication for LP (spinal tap) is a headache.	Pre	20	14	65	0	**<0.001**
	Post	71	16	12	0	
Meningitis can be diagnosed via LP.	Pre	59	41	N/A	**N/A**	**<0.01**
	Post	84	16	N/A	N/A	
Multiple sclerosis can be diagnosed via LP.	Pre	67	33	N/A	**N/A**	0.13
	Post	78	22	N/A	N/A	
Depression can be diagnosed via LP.	Pre	4	96	N/A	N/A	0.61
	Post	8	92	N/A	N/A	
Stroke can be diagnosed via LP.	Pre	12	88	N/A	N/A	0.55
	Post	18	82	N/A	N/A	
Skin diseases can be diagnosed via LP.	Pre	12	88	N/A	N/A	0.22
	Post	18	82	N/A	N/A	
Parkinson's disease can be diagnosed via LP.	Pre	41	59	N/A	N/A	>0.05
	Post	39	61	N/A	N/A	

Bold values are statistically significant, *p* < 0.05.

Participants who did not have a previous LP also significantly improved in their knowledge of what an LP procedure entailed. These participants also accurately identified frequent side effects and rare side effects. There was no significant change in their ability to identify what an LP could be used to diagnose.

### Willingness to undergo an LP

Table 6 demonstrates the results of preferences and willingness to undergo an LP before and after watching the video. There was no significant difference in participants' willingness to undergo an LP for direct benefits to them, including early diagnosis of memory problems and deciding upon clinical treatment plans. There was also no significant difference in participants' willingness to undergo an LP for indirect benefits to them, including determining future potential for a disease, improving knowledge of a disease the participant might have, or improving HIV research. There was also no significant difference, on a separate question, on participants' willingness to undergo an LP when it had no direct benefit to them for improving HIV research. There was no significant difference in the number of participants, pre- and post-video, who would never undergo an LP under any circumstance.

**Table 6:** Preference for LP pre- and post-LP educational video among all participants (*N* = 99).

Question		Yes (%)	No (%)	*p*-value
I would consider an LP if my doctor can diagnose memory problems I may have at an early stage.	Pre	37	62	0.15
	Post	48	51	
I could consider an LP if it is necessary for my doctor to decide on treatment for a disease I have.	Pre	64	35	>0.99
	Post	65	34	
I would consider an LP if my doctor can determine the chance of me developing a disease in the future.	Pre	41	58	0.25
	Post	50	49	
I would consider an LP if it improves knowledge on a disease I have.	Pre	37	62	0.11
	Post	49	50	
I would consider an LP if it would improve HIV research.	Pre	50	49	0.15
	Post	61	38	
I will not have an LP under any circumstance.	Pre	4	95	>0.99
	Post	5	94	
I would have an LP to contribute to research on HIV, even if it would not help me directly.	Pre	63	24	0.63
	Post	65	26	

## Discussion

Our study has demonstrated that PWH had improved knowledge of LPs after watching an educational video on an LP procedure. However, watching the LP educational video did not appreciably affect PWH's preferences for considering having an LP done. These findings represent the first study to assess LP knowledge and preference for undergoing the procedure in a group of middle-aged and older PWH before and after watching a patient-centered educational video.

We chose this specific educational video based on the combination of its duration (5 min) and easy-to-understand patient language. The video comes from an online library for providers consisting of videos geared toward patient education, and as such, we found it to be most conducive to the goals and methods of our study (9). The video primarily utilizes infographics and 3D animations to discuss the procedure's indications, diagnostic uses, and demonstrate the procedure itself. Although the influence of 3D animations in our participants' choices is difficult to assess, it is possible that the use of 3D animations, compared to a live demonstration of the procedure, reduced the vividness of undergoing an LP in subjects' minds. Conversely, utilizing 3D animations may have also decreased subjects' ability to link the animation with what they would undergo in real life.

In order to assess knowledge about an LP, it is important to ask patients about their willingness to have this procedure done for research purposes (12, 10). Showing patients a video providing visual representations of the procedure that explains the risks involved with the procedure has been shown to mitigate fears and anxiety surrounding an LP in patients with Alzheimer's disease (7), making it more likely that patients will be well-informed and participate in studies with the goal of advancing neurological research among aging PWH. In one study from Saudi Arabia, only 19.2% of participants who had never had an LP had knowledge of what an LP was (13), but research is lacking from the U.S. In our study, after removing participants who had had an LP previously, we found that 83.7% did have appropriate knowledge of what an LP was prior to watching the video, highlighting that procedure knowledge is high in our specific population.

LPs are important in the context of HIV for diagnosing CNS opportunistic infections or co-infections that could improve diagnostic certainty and help guide clinical management. HIV-associated neurocognitive disorder (HAND) is a common chronic complication of HIV resulting in delayed processing of information and impairments in attention and working memory (14), HAND is estimated to affect 43% of PWH worldwide, yet its pathophysiology remains unknown (15). Research investigating the pathophysiology of HAND and the role of inflammation in the CNS compartment that may lead to neurodegeneration is of increasing importance and requires LPs to access spinal fluid for further investigation of important inflammatory, immune activation and neurodegeneration biomarkers and to develop novel treatments to help reduce the burden of HAND as PWH age (16, 17). In our study, we did not find that showing an educational video on LP procedures helped improve willingness to undergo an LP for HIV research purposes nor did anxiety levels surrounding the LP procedure diminish after watching the video. This highlights that stigma and fear surrounding the LP procedure may not improve even when LP knowledge improves.

LPs are known to be associated with significant stigma, particularly in sub-Saharan Africa and other low to-middle-income regions. Negative outcomes, including death or high morbidity has been historically perceived by patients to be caused by an LP. In Zambia, one qualitative study identified several patient-related factors that led to barriers to LP, including: lack of understanding of what cerebrospinal fluid is, misinformation about LPs, mistrust of doctors, fear of blame and peer pressure from family members or friends against consenting for an LP (18). Similar to our study, another study in Zambia found that in-person counseling and education on LP procedures was insufficient to increase uptake of the LP procedure and that pressure from family members to not consent for LP was more important than the knowledge gained during provider counseling (19). In China, a study found that factors associated with a positive attitude toward LPs for the diagnosis of Alzheimer's disease included younger age, higher educational level and higher income (20). Further research, including qualitative studies with in-depth interviews or focus groups assessing reasons for willingness and unwillingness to undergo LPs is needed to further improve neurological research among PWH.

Beyond LPs, PWH may sometimes require additional screening procedures such as pap smears or anal colposcopies that can be perceived as discomforting or frightening to undergo. Using educational videos to demonstrate the procedures, as we have in our study, may improve clinical care for PWH by addressing these anxieties and increasing awareness of routine preventative procedures. One study conducted in Ghana, for example, demonstrated that participants who received video education were significantly more aware of what cervical cancer was, as well as more likely to undergo pap smears for cervical cancer screening (21). Video education has been demonstrated across several studies to significantly reduce anxiety for participants undergoing cervical colposcopy (22, 23). Another study in Puerto Rico, assessing willingness to participate in a clinical trial surrounding anal cancer treatment, found increased willingness among individuals who received video education to undergo pre-screening and examination for the trial (24). Although our study did not demonstrate that PWH were more willing to undergo an LP for either diagnostic or research purposes, this may not be the case for procedures for preventative healthcare and needs to be ascertained in PWH.

One potential benefit demonstrated by our study results is that video education can improve patients' knowledge of diagnostic procedures without unduly influencing their decisions regarding whether to undergo such procedures. This is demonstrated by the fact that our study demonstrated increased knowledge of an LP, without a concurrent increase in willingness to undergo an LP. Several factors not measured as part of this study (such as emotional impact behind deciding on undergoing an LP for research purposes) may also play a role, and perhaps this would be measured with qualitative study in the future. Future studies seeking to assess the influence of video education on participants' willingness to undergo diagnostic procedures may attempt to combine video education with standardized in-person education by a provider, and compare that to a cohort only receiving in-person education.

Our study has limitations worth noting. Our sample size was small (99 subjects). We conducted quantitative assessments of participants' views, and further qualitative interviews would enable additional understanding of participants’ views regarding LPs. We did not collect data on chronicity of diseases for which patients may receive a lumbar puncture. The specificity of our study population, all of whom were PWH age ≥ 50 years of age, may potentially limit the generalizability of our findings to a population of participants without HIV or those who are younger than 50 years. The majority of our study sample consisted of African American subjects, which may limit our sample's representativeness of the general demographic population of the country. Moreover, all participants were virally suppressed and presumably adherent with antiretroviral therapy as part of the parent study eligibility criteria, which may not represent all PWH.

## Conclusions

We found that, while LP knowledge among PWH significantly improved, even among those who had never had an LP, willingness to undergo an LP did not. Other educational interventions, such as in-person demonstrations or models, may help mitigate fear of LP. Our study provides important insight into the knowledge and perceptions of PWH when asked to undergo an LP and demonstrates that video-based education may not be sufficient to mitigate fears surrounding LP procedures. Our study demonstrates that video education improves knowledge without unduly influencing patient decision-making Future studies would determine whether willingness to undergo a lumbar puncture impacts the decision to undergo a lumbar puncture for research, and whether a face-to-face explanation by the provider or other learning modalities may improve willingness and decision to undergo a lumbar puncture more so than watching a video. Developing qualitative studies to assess reasons for fears or unwillingness to participate in research involving LPs or obtain an LP for clinical purposes, even if required for diagnostic or treatment purposes, is needed. This is key for advancement of recruitment into research on HAND among aging PWH.

## Data Availability

The raw data supporting the conclusions of this article will be made available by the authors, without undue reservation.
